# Downregulation of PHEX in multibacillary leprosy patients: observational cross-sectional study

**DOI:** 10.1186/s12967-015-0651-5

**Published:** 2015-09-11

**Authors:** Sandra R. Boiça Silva, Ximena Illarramendi, Antonio J. Tempone, Pedro H. L. Silva, José A. C. Nery, Alexandra M. V. Monteiro, Maria Cristina V. Pessolani, Edson Boasquevisque, Euzenir N. Sarno, Geraldo M. B. Pereira, Danuza Esquenazi

**Affiliations:** Laboratório de Hanseníase, Instituto Oswaldo Cruz (IOC) – Oswaldo Cruz Foundation (FIOCRUZ), Av. Brasil 4365, Manguinhos, Rio de Janeiro, RJ 21040-360 Brazil; Laboratório de Microbiologia Celular, Instituto Oswaldo Cruz (IOC) – Oswaldo Cruz Foundation (FIOCRUZ), Av. Brasil 4365, Manguinhos, Rio de Janeiro, RJ, 21040-360 Brazil; Laboratório de Biologia Molecular de Parasitas e Vetores, Instituto Oswaldo Cruz (IOC) – Oswaldo Cruz Foundation (FIOCRUZ), Av. Brasil 4365, Manguinhos, Rio de Janeiro, RJ, 21040-360 Brazil; Disciplina de Patologia Geral e Laboratório de Imunopatologia, Departamento de Patologia e Laboratórios, Faculdade de Ciências Médicas, Universidade do Estado do Rio de Janeiro (UERJ), Av Professor Manuel de Abreu 444, Vila Isabel, Rio de Janeiro, RJ, 20550-170 Brazil; Serviço de Medicina Nuclear, Departamento de Radiologia, Faculdade de Ciências Médicas, Universidade do Estado do Rio de Janeiro (UERJ), Av. Prof. Manuel de Abreu 444, Vila Isabel, Rio de Janeiro, RJ, 20550-170 Brazil

**Keywords:** PHEX, *Mycobacterium leprae*, Leprosy, Vitamin D, Bone damage, Inflammatory cytokines

## Abstract

**Background:**

Peripheral nerve injury and bone lesions, well known leprosy complications, lead to deformities and incapacities. The phosphate-regulating gene with homologies to endopeptidase on the X chromosome (*PHEX*) encodes a homonymous protein (PHEX) implicated in bone metabolism. PHEX/*PHEX* alterations may result in bone and cartilage lesions. *PHEX* expression is downregulated by intracellular *Mycobacterium leprae* (*M. leprae*) in cultures of human Schwann cells and osteoblasts. *M. leprae* in vivo effect on PHEX/*PHEX* is not known.

**Methods:**

Cross-sectional observational study of 36 leprosy patients (22 lepromatous and 14 borderline-tuberculoid) and 20 healthy volunteers (HV). The following tests were performed: PHEX flow cytometric analysis on blood mononuclear cells, cytokine production in culture supernatant, 25-hydroxyvitamin D (OHvitD) serum levels and ^99m^Tc-MDP three-phase bone scintigraphy, radiography of upper and lower extremities and blood and urine biochemistry.

**Results:**

Significantly lower PHEX expression levels were observed in lepromatous patients than in the other groups (χ^2^ = 16.554, *p* < 0.001 for lymphocytes and χ^2^ = 13.933, *p* = 0.001 for monocytes). Low levels of 25-(OHvitD) were observed in HV (median = 23.0 ng/mL) and BT patients (median = 27.5 ng/mL) and normal serum levels were found in LL patients (median = 38.6 ng/mL). Inflammatory cytokines, such as TNF, a *PHEX* transcription repressor, were lower after stimulation with *M. leprae* in peripheral blood mononuclear cells from lepromatous in comparison to BT patients and HV (χ^2^ = 10.820, *p* < 0.001).

**Conclusion:**

Downregulation of PHEX may constitute an important early component of bone loss and joint damage in leprosy. The present results suggest a direct effect produced by *M. leprae* on the osteoarticular system that may use this mechanism.

**Electronic supplementary material:**

The online version of this article (doi:10.1186/s12967-015-0651-5) contains supplementary material, which is available to authorized users.

## Background

Bone disease occurs in many clinical conditions, from infectious to metabolic, inflammatory, cancer and genetic disorders. Among the infectious diseases, leprosy, caused by *Mycobacterium leprae*, is widely known because of the acral deformities. Lepromatous leprosy (LL) is one of the polar forms of the spectrum of the disease, in which patients exhibit high bacillary load, numerous skin lesions and hypo-responsiveness to *M. leprae*. At the opposite end of the spectrum, the polar tuberculoid patients (TT) and borderline tuberculoid (BT) show reduced bacillary load (undetectable in slit-skin smear), few cutaneous lesions and pathogen-specific cellular immune response [1].

The prevalence of bone damage is variable according to the casuistic evaluated. In a retrospective cohort of 105 newly-diagnosed adult multibacillary leprosy patients surveyed for up to 8 years after release from treatment, 23 % of the patients were found to have acral resorption [2]. Also, in patients with established disability, bone damage was found to be present in 80 % of patients [3]. The small bones of hands and feet are the most affected, followed in frequency by facial bones [4, 5]. In the affected bones, *M. leprae*-filled macrophages can be observed in the medullar cavity and trabecular bone destruction [4]. Signs of periostitis, different degrees of phalange resorption, bone cysts, trabeculation loss and osteoporosis can be observed in image studies [3, 6].

The X-linked hypophosphatemic rickets is a rare disease that produces deficient bone matrix mineralization resulting in rickets or osteomalacia, hypophosphatemia, and change**s** in vitD metabolism [7]. It is caused by the absence of phosphate-regulating gene with homologies to endopeptidase on the X chromosome (*PHEX*) [8].

How can these two diseases be related? Reduction of *PHEX* transcription and protein expression (PHEX) was found to be produced by *M. leprae* in cultured Schwann cells and human osteoblasts [9]. PHEX binds to the small integrin-binding ligand, N-linked glycoprotein (SIBLING) family of proteins and participates in a complex pathway that modulates bone matrix mineralization, phosphate renal excretion, serum levels of fibroblast growth factor 23 (FGF23) and 1,25(OH)_2_vitamin D regulation [10–20]. Could this mechanism induce bone damage in lepromatous patients? To answer this question we evaluated PHEX expression, its possible regulatory mediators, and its relationship with bone disease in a group of patients with LL.

## Methods

### Study population

**Fig. 1:**
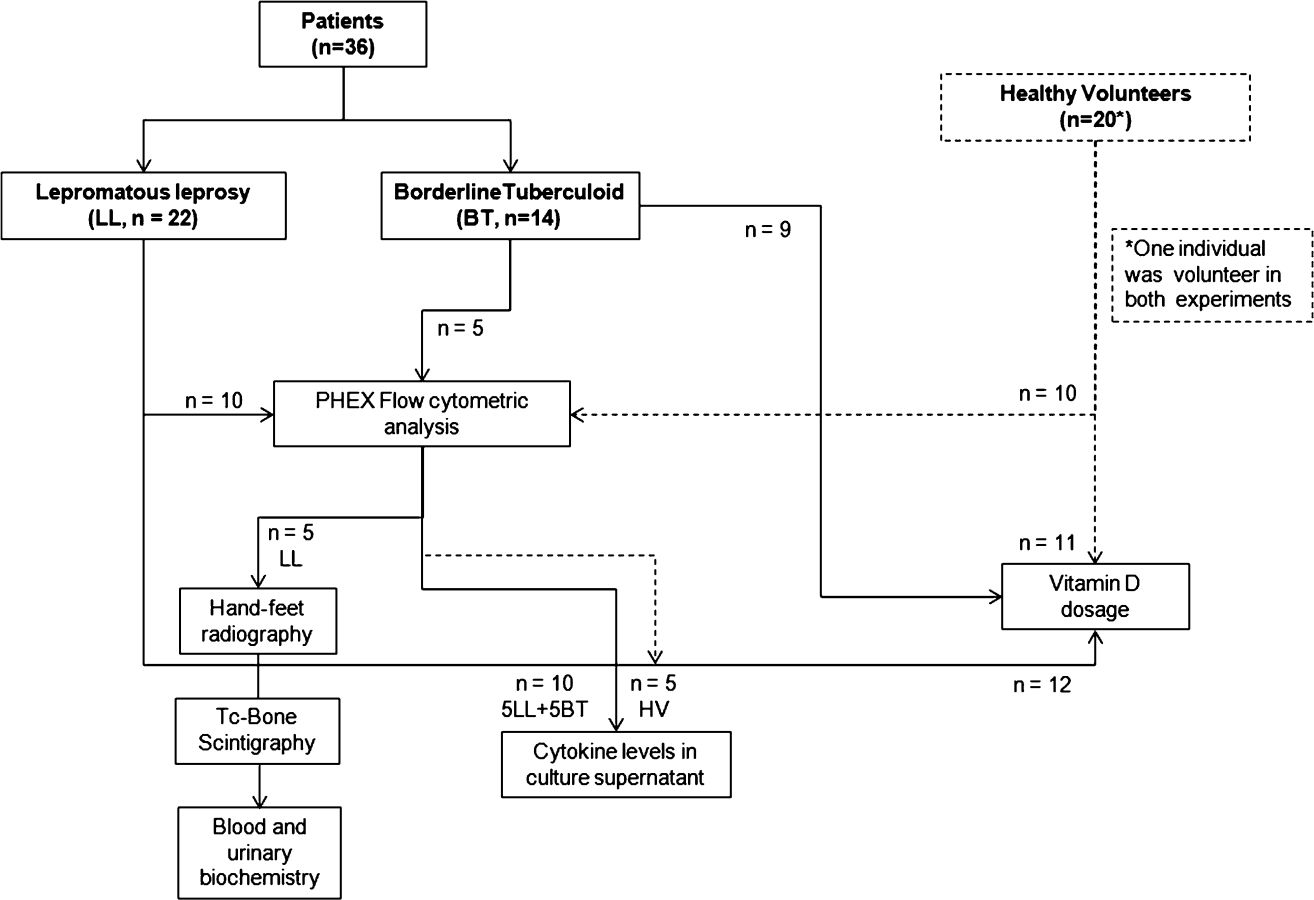
Study design. Groups of individuals evaluated and tests performed. A total of 36 patients and 20 healthy volunteers were clinically and/or laboratory assessed

This case series study was developed at the Leprosy Laboratory and Souza Araújo Outpatient Unit of Oswaldo Cruz Institute, Fiocruz, and the Santa Casa de Misericórdia Hospital, in Rio de Janeiro, Brazil. A total of 36 newly-diagnosed untreated patients (22 LL and 14 BT) were evaluated. In addition, 20 healthy volunteers (HV), selected from medical and laboratory staff were included as negative controls for the assessment of the immune response against *M. leprae* and VitD measurements (Fig. 1).

The leprosy patients followed routine examinations and were classified according to the Ridley and Jopling criteria [1]. The disability grade resulting from the presence of neurological signs and symptoms in the patients’ eyes, hands and feet was registered by a physiotherapist [21]. The bacillary index (BI) was obtained from the slit-skin smear of six sites. The project was approved by the Oswaldo Cruz Foundation Research Ethics Committee, protocol number 205/03.

### In vitro tests

Peripheral blood mononuclear cells (PBMC) from LL, BT and HV were isolated over ficoll-hypaque (GE Healthcare, UK) by density gradient centrifugation, washed in PBS, and divided into two parts, one for the flow cytometry study, the other part for the cytokine production assay.

#### Flow cytometry

In order to detect PHEX surface expression, PBMCs were re-suspended in flow cytometry buffer and incubated with anti-PHEX purified followed by a fluorescein isothiocyanate labeled polyclonal antibody (FITC; Alpha Diagnostic International, Inc., USA) as secondary marker. For T cell and monocyte identification, the cells also were marked with anti-CD3-phycoerythrin and anti-CD14-cychrome monoclonal antibodies (BD Biosciences, USA). Following incubation, the cells were analyzed in a FACSCalibur flow cytometer (BD Biosciences, USA), as described [22].

#### Cytokine production assay

PBMC were re-suspended in the AIM-V culture medium (10^6^ cells/mL), and cultured for 5 days in 96-well round-bottom culture plates (2 × 10^5^ cells/well; Corning Inc. Life Sciences, USA). TNF, IL-1β and IFN-γ levels were measured in the culture supernatants collected from unstimulated and 20 µg/mL whole-irradiated *M. leprae*-stimulated cultures (provided from Colorado State University, USA through the NIH/NIAID “Leprosy Research Support” contract N01 AI-25469) and 1 μg/mL Staphylococcal Enterotoxin B (SEB; Sigma Chemical Co., St. Louis, MO, USA). The cytokines levels were measured using a multiplex kit (Lincoln-Millipore, EMD Millipore, USA) and a Luminex 100 analyzer (Luminex 1.7 software; Luminex Corporation, USA).

### Complementary exams

#### Image studies

The hands and feet of a subgroup of 5 LL patients were studied by conventional X-ray. In addition, computed radiography digital X-ray antero-posterior skiagrams were obtained of the hands to look for more detailed bone changes. The radiographs were analyzed by two experienced radiologists for the presence of specific and non-specific bone alterations. Findings were decided in consensus. Radiological bone resorption was defined as partial or total loss of one or several phalanges or metatarsal or carpal bones; specific osteolysis was defined as a break in the contour (cortex and subcortical areas of the epiphyses) of the distal phalanx into bushy and berry-like lesions, and concentric absorption (longitudinal loss of the diaphysis) and distal absorption as non-specific osteolysis. Three-phase bone scintigraphy was performed after intravenous injection of 555–1110 mBq (15–30 mCi) of ^99m^Technetium-methylene diphosphonate (^99m^Tc-MDP) using a low-energy, high-resolution collimator (E.CAM, Siemens) in the same five patients. After injection of the radiopharmaceutical, 2-s images of the hands were immediately obtained in a 64 × 64 matrix. Blood pool images of both the hands and feet were acquired 5–10 min after administration of ^99m^Tc-MDP and stored in a computer via a 128 × 128 matrix. Delayed anterior and posterior whole body images were used to determine the extent and distribution of the alterations. The third-phase images were acquired between 3–5 h and included spot images of the hands, feet, and other regions with alterations in the WB images. All spots were acquired in about 4–5 min using a 256 × 256 matrix.

#### Laboratory analysis

Venous blood and 24-h urine samples were collected in these LL patients. The serum levels of alkaline phosphatase, albumin, calcium, phosphorus, parathyroid hormone (PTH), parathyroid hormone-related protein (PTHrP), pro-collagen type I, osteocalcin, and 1,25(OH)_2_vitamin D (high performance liquid chromatography) were measured. The levels of calcium, phosphorus, protein, N-telopeptide, pyridinoline and desoxypyridinoline were evaluated in the urine samples. Serum samples were obtained from additional 32 individuals (12 LL, 9 BT and 11 HV) for determination of 25-vitD levels by quimioluminescence. This group of subjects were included after the first five LL patients were evaluated because low levels of this vitamin D3 metabolite is considered a risk factor for the development of various diseases and, in order to reduce the effect of environmental variables, the same time interval was needed.

### Statistical analysis

The statistical analysis was done using SPSS^®^ 16. Due to the small sample number and non-gaussian distribution, nonparametric tests were used to compare variables, including outliers and extreme values. Significance level was established at 95 %. The differences in the levels of cytokines, 25-(OH) vitD and PHEX expression were evaluated using the Kruskal–Wallis Chi square (KW-χ^2^), and the Mann–Whitney test. Graphs were done using the Prism 6.0 software (GraphPad Software Inc., USA).

## Results and discussion

Fourteen BT patients (nine males) had a mean age of 37 years (range 15–58 years) and negative BI, except for one patient with 0.25log. Twenty two LL patients (17 male) were in average 50.3 years old (from 29–82 years) had a BI ranging from 1.25 to 6.0 (median = 4.25). The twenty one HV were young adults (six males) from 19 to 52 years old (mean = 31 years).

### Ex vivo study: PHEX expression in PBMC and cytokine production

**Fig. 2:**
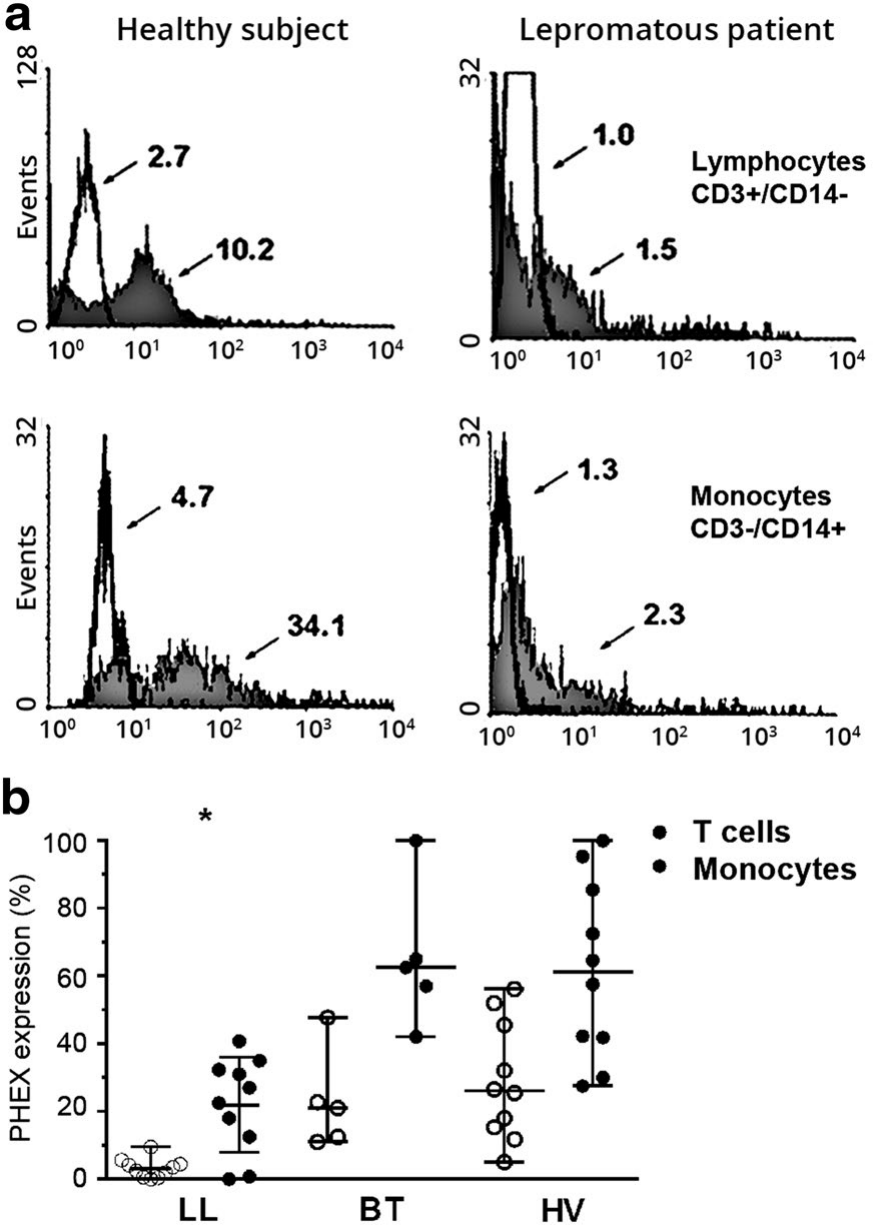
PHEX expression in T lymphocytes and monocytes by flow cytometric analysis. **a** Example of a flow cytometry result comparing PHEX protein expression in lymphocytes (*upper*) and monocytes (*lower*) of a healthy volunteer (*left*) a lepromatous patient (*right*). The *arrows* show that the fluorescence median values were lowest in the patient. **b** Flow cytometry analysis of peripheral blood leukocytes of LL (n = 10), BT patients (n = 5) and healthy volunteers (n = 10) showing a decrease in PHEX protein expression in LL patients (*p < 0.05)

**Fig. 3:**
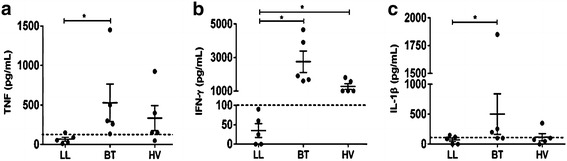
Multiplex analysis of TNF (**a**), IL-1β (**b**) and IFN-γ (**c**) levels in 5-day culture supernatants. The results reflect cytokine levels in *M. leprae*-stimulated cultures subtracted from unstimulated cultures. The three inflammatory cytokines were significantly lower in LL patients (n = 5) compared to BT patients (n = 5; *p < 0.05). All cytokines were produced in response to SEB (data not shown). IFN-γ levels in LL patients were also significantly lower in comparison to healthy volunteers (n = 5; *p < 0.05). *Dotted-lines* indicate established positive production levels in relation to negative controls

PHEX expression was significantly lower in LL as compared to BT patients and HV (Fig. 2) in both monocytes (KW-χ^2^ = 13,933, *p* = 0.001) and lymphocytes (KW-χ^2^ = 16,554, *p* < 0.0001). This observation suggests a negative modulation of PHEX expression by the large quantity of mycobacteria present in LL patients. Once the downregulation of PHEX expression was observed, we sought to determine the production of inflammatory cytokines by *M. leprae*-stimulated blood leukocytes. TNF is known to modulate the *PHEX* transcription [23]. In addition, TNF intervenes in the host defense against *M. leprae*, and is low in LL patients [24, 25]. Cytokine production by blood leukocytes in response to *M. leprae* was detected in most LL and BT patients’ and HV’s samples (Fig. 3).

Due to specific *M. leprae* hypo-responsiveness in LL patients, the expected low production levels of pro-inflammatory cytokines were observed in these patients. TNF and IFN-γ levels were significantly lower in LL patients as compared to the other two groups (KW-χ^2^ = 10.820, *p* < 0.001; KW-χ^2^ = 9.397, *p* = 0.003), and IL-1β production was similar in the three groups (KW-χ^2^ = 0.483, *p* = 0.799). As expected, all SEB-stimulated cells (positive control) demonstrated high levels of TNF, IL-1β and IFN-γ production (data not shown). Therefore, the observed reduced PHEX expression in this set of LL patients was not related to the in vitro TNF production of the *M. leprae*-stimulated blood leukocytes. This finding suggests that TNF may not be involved in the PHEX downregulation observed in LL patients.

### In vivo study

**Fig. 4:**
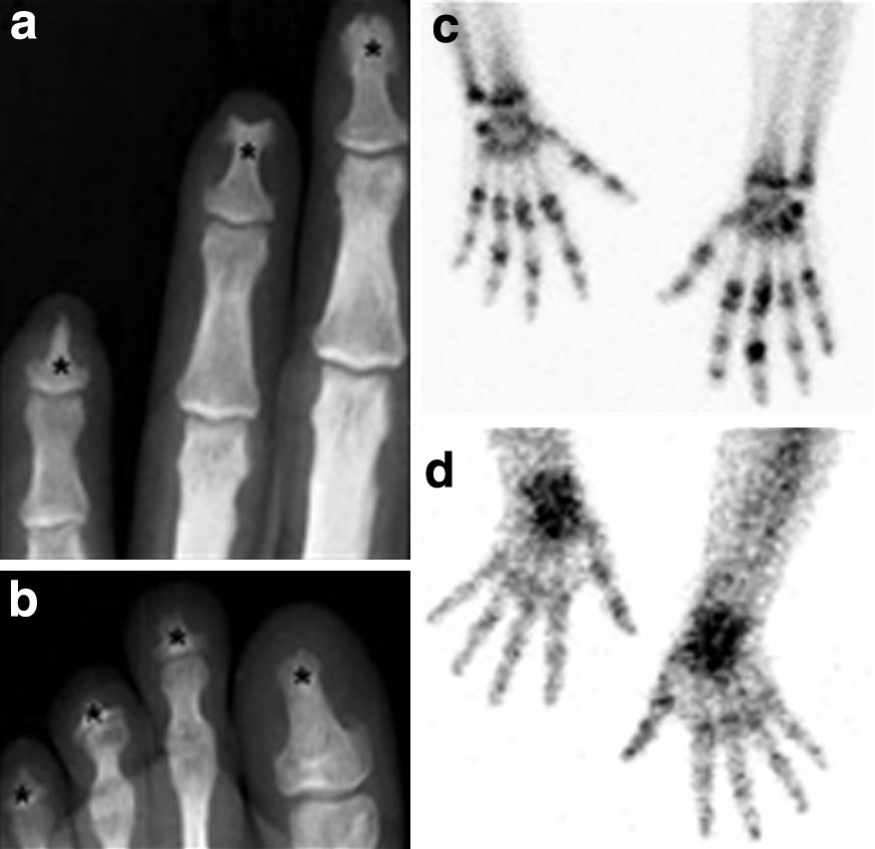
Newly diagnosed lepromatous patients exhibits bone and cartilage lesions. **a**, **b** Radiography of left hand and foot. *Asterisks* indicate the distal phalanx erosion, typical hallmark of leprosy. **c** Third-phase bone scintigraphy image shows increased bone uptake of ^99m^Tc-MDP in both hands. Hyper-fixation occurred in bones and joints of phalanges, metacarpus and wrists. Important joint alterations were evidenced in all patients. **d** The early phase scintigraphy shows localised radiotracer activity on the wrists

Five LL patients (19–37 years old) were selected for clinical, radiological, bone scintigraphy and metabolic evaluation. Young adults were selected to exclude bone or joint changes as well as laboratory alterations due to the aging process. Three patients, all with BI higher than 4.0, had radiological changes and bone resorption (Additional file 1: Table S1). First-phase scintigraphy was abnormal in 4 of the patients, who showed blood flow asymmetry and blood-pool alterations in the lower and upper extremities. Increased bone uptake of ^99m^Tc-MDP was found in the hands or feet of all five patients. A bilateral involvement was observed but the right extremities were more frequently affected. The distal phalange alterations demonstrated by X-ray imaging (Fig. 4a, b) suggest long-term resorption thus ^99m^TC-MDP uptake is reduced in these areas (Fig. 4c). However, in other areas mainly the joints, hyper-fixation indicated more recent activity (Fig. 4c, d). Important joint alterations were also evidenced in all patients. Increased uptake was seen on the interphalangeal joints, distal phalanges of the hands, metacarpal bones and/or wrists. The metacarpophalangeal and metatarsophalangeal joints were the most severely affected, followed by the intercarpal and the proximal interphalangeal articulations in the hands. All patients presented normal serum levels of alkaline phosphatase, albumin, calcium, phosphorus, 1,25(OH)_2_vitamin D, PTHrP, in addition to normal urinary calcium, phosphorus, and protein levels. Parathyroid hormone dosage was normal (7–53 pg/mL) in all but one patient (91 pg/mL, Additional file 1: Table S1, patient A). This patient also exhibited elevated blood levels of type I procollagen (162 ng/mL) and osteocalcin (68.8 ng/mL), including higher levels of N-telopeptide in the urine (244 nmBCE/nmol). The levels of pyridinoline and deoxypyridinoline in the urine were also the most elevated among the patients, indicating a high-turnover bone disease. Missing samples or shorter time interval for the 24-h urine collection were suspected in patients C and D, taking into account their low levels of urinary creatinine excretion. Despite the missing samples in the 24-h urine collection, the elevation of the urinary collagen degradation products, still suggested increased bone resorption in these patients, as shown by the radiography and scintigraphy images (Additional file 1: Table S1). Interestingly, patients with higher levels of PHEX exhibited no alterations in radiographies, and fewer alterations in scintigraphy than patients with low PHEX expression.

Low vitD levels are found in a wide range of the world population. In Brazil it has been observed in about 60 % of the adolescent population [26] and 80 % of the adults in the Amazon region [27]. This vitamin is important for the immune system. It participates in the defense against mycobacteria by stimulating cathelicidin synthesis, both in macrophages and in dendritic cells [28, 29] as well as in keratinocytes [30]. Inhibition of the vitD-dependent microbicidal pathway has been shown in LL patients [31]. Therefore, we hypothesized that leprosy patients, especially those with LL form, would have vitD deficiency, because of their reduced cellular response against *M. leprae*. However, we only found two patients with levels within the insufficiency range and two with vitamin deficiency (<20 ng/mL). Taking the value of 30 ng/mL as the lower normal serum value, no deficit was observed in the group of LL patients (mean = 35.3 ± 12.7 ng/mL, 95 % CI 27.19–43.36). BT patients had around normal levels of vitD (mean = 29.4 ± 10.6 ng/mL, 95 % CI 21.22–37.49), but six patients were classified as insufficient and one was considered deficient. Interestingly, only 3 out of the 11 HV (27 %) had normal vitD serum levels (mean = 23.5 ± 8.4 ng/mL, 95 % CI 17.85–29.13), four had insufficient levels and four were in the deficient range. This finding was similar to other studies in which high percentages of vitD insufficiency/deficiency in healthy controls, especially those from medical or laboratory staff [32]. Also, no difference in 25-(OH) vitD levels has been observed in patients with multibacillary leprosy as compared to healthy individuals [33].

As a whole, the patients had significantly higher vitD levels (χ^2^ = 6.774, *p* = 0.034) than the HV. Although at the onset of infection, low host vitD levels could be advantageous for *M. leprae*, impairing the vitD-dependent microbicidal pathway, once the mycobacteria escape the host’s initial response, and long-term persistence in macrophages and Schwann cells is established, the presence of vitD could then hinder the elimination of pathogens due to its immunomodulatory effects. VitD could participate in the change of the response of Th1 lymphocytes to Th2 cells, and in the modulation of the macrophage differentiation from M1 to M2 [31, 34].

Bone damage has for long been observed in LL patients [4, 35]. It has been attributed to the effect of abnormal serum electrolytes, peripheral neuropathy, local trauma, inflammation, hypogonadism [36], prolonged rest and malnutrition, or prolonged use of glucocorticoids to control reactional episodes [37, 38]. In the present study, the LL patients showed no detectable differences in serum electrolytes in comparison to the BT patients, had no evidence of malnutrition and had not been treated with corticosteroids. Similarly, bone disease has been observed in leprosy patients with none of these risk factors [2]. Which mechanism could explain bone disease in such patients?

Peripheral neuropathy is a common finding in leprosy because of the *M. leprae* tropism to infect Schwann cells. It is more common in multibacillary than in paucibacillary patients [39]. Some degree of peripheral neuropathy was observed in the five patients evaluated with radiography and scintigraphy, two had hand, feet and/or facial hypo or anaesthesia, and three had deformities or permanent disability. However, this finding could not explain all of the bone and joint lesions observed in these patients. Bone changes in patients with disabilities and deformities have characteristic features not observed in the five patients [3].

The physiopathology of peripheral neuropathy as a cause of acral deformities is well explained by the Charcot neuro-arthropathy, which is frequently seen in longstanding diabetic patients [40]. This factor clearly explains deformities of the lower extremities, which are under the influence of body weight and locomotion micro-traumas. However, leprosy patients have bone and joint damage in upper extremities and the face, which are not observed in the diabetic patients, who suffer from glove and stocking neuropathy. In addition, specific bone changes have been found to be significantly increased in patients with absent or incomplete anti-leprosy treatment [3]. Interestingly, in a murine model of *M. leprae*, the infection directly caused bone damage in the infected mice that had bilateral rear-limb palsy [41].

PTHrP, PTH [42] and 1,25(OH)_2_vitamin D [43, 44] produce negative regulation of *PHEX* transcription. However, we found no change in serum PTHrP or 1,25(OH)_2_vitamin D in the subgroup of patients evaluated, and only one of the patients had increased levels of parathyroid hormone. TNF is another negative modulator of *PHEX* [45] and is a known risk factor for decreased bone mineral density in chronic inflammatory bowel disease [23]. The stimulation of PBMC by *M. leprae* in cell cultures did not result in increased production of TNF.

Once the inflammatory mediator production in response to *M. leprae* or vitD deficiency could not contribute to the altered PHEX expression in the patients evaluated, a direct effect of *M. leprae* becomes a plausible cause. The downregulation of PHEX can contribute to the osteoarticular lesions observed in patients, as it would result in greater amounts of ASARM peptides able to directly inhibit the mineralization of the bone matrix [10, 11, 15]. In addition, PHEX decay and increase of ASARM peptides would favor the survival of osteocytes, inhibiting surrounding mineralization [18]. This would be advantageous to *M. leprae* survival, allowing the maintenance/proliferation of infected cells in bone, where previous studies have found bacilli [46].

## Conclusions

The present was an exploratory study to evaluate a possible role of PHEX in bone alterations in leprosy, already shown in other diseases [10, 11, 15, 18]. Based on our previous findings on the negative modulation of PHEX in human osteoblast lineage [9], and the observed in blood leukocytes obtained from patients with lepromatous leprosy, we suggest that PHEX downregulation may be a possible mechanism by which *M. leprae* directly affect bone. Bone alterations may be present in leprosy patients with none of the known risk factors of bone disease. There is still an extensive amount of unknown matters regarding this complication in leprosy, as well as the role of PHEX in bone homeostasis that still need to be addressed. We looked into one of the possible mechanisms hoping to find an additional pathway that may help to explain bone disease physiopathology in leprosy.

## Additional file

Additional file 1:
**Table S1.** Clinical and laboratory findings of five LL patients.
