# Supplementary material for: Downregulation of PHEX in multibacillary leprosy patients: observational cross-sectional study
Source: J Transl Med. 2015 Sep 11;13:296. doi: 10.1186/s12967-015-0651-5 (PMC4566286; doi:10.1186/s12967-015-0651-5)
Supplement: Additional file 1: — Table S1. Clinical and laboratory findings of five LL patients. [file 12967_2015_651_MOESM1_ESM.doc]

**Table S1.** Clinical and laboratory findings of five LL patients.

| **ID** | **Sex** | **Age** | **BMI** | **BI** | **GD** | **Edema** | **X-Ray** | **STG** | | **Pyrid** | **Desoxyp** |
| --- | --- | --- | --- | --- | --- | --- | --- | --- | --- | --- | --- |
|  |  |  |  |  |  |  |  | **Extremities** | **Face** |  |  |
| A | M | 19 | 22.9 | 4.0 | 2 | HF bilat | Resorption,  demineralization | +++ | ++ | ↑↑ | ↑↑ |
| B | M | 26 | 33.2 | 4.3 | 2 | RH | Resorption,  bone cyst | +++ | ++ | ↑ | WRV |
| C | F | 20 | 32.9 | 5.0 | 1 | HF bilat | Lysis | +++ | + | ↑ | ↑ |
| D | F | 36 | 21.0 | 2.2 | 2 | HF bilat | No alterations | ++ | No uptake | WRV | ↑ |
| E | F | 37 | 19.5 | 4.8 | 1 | F bilat | No alterations | + | No uptake | WRV | ↑ |

ID: patient identification; BMI: body mass index; BI: bacilloscopic index; GD: grade of disability 1=hand/feet anaesthesia, 2=deformity/permanent disability; STG: scintigraphy; Pyrid: Pyridinoline; Desoxyp: Deoxypyridinoline; H: hands; F: feet; R: right; ↑ above normal range; ↑↑ twice above normal range; +++ intense ^99m^Tc-MDP uptake; ++ moderate ^99m^Tc-MDP uptake; + slight ^99m^Tc-MDP uptake; WRV= within reference values.
